# Atrial Fibrillation as an Initial Presentation of Apathetic Thyroid Storm

**DOI:** 10.7759/cureus.17786

**Published:** 2021-09-07

**Authors:** Hassam Ali, Shiza Sarfraz, Lareb Hassan, Hadeera Ali

**Affiliations:** 1 Internal Medicine, East Carolina University, Vidant Medical Center, Greenville, USA; 2 Anesthesiology, Bahawal Victoria Hospital, Quaid-E-Azam Medical College, Bahawalpur, PAK; 3 Medicine and Surgery, Quaid-e-Azam Medical College, Bahawalpur, PAK; 4 Internal Medicine, CMH Institute of Medical Sciences, Bahawalpur, PAK

**Keywords:** graves´disease, atrial fibrillation, apathetic hyperthyroidism, thyroid-storm, thyrotoxicosis

## Abstract

Atrial fibrillation as an initial presenting symptom of an apathetic thyroid storm is under-reported, especially in the setting of undiagnosed hyperthyroidism. Very rarely, thyroid storm can present with apathetic symptoms. The author presents a case of apathetic thyrotoxicosis with atrial fibrillation. The patient had a generalized weakness, lethargy, and weight loss as initial symptoms and was found to have atrial fibrillation, which was initially thought to be the inciting event. However, further evaluation revealed a new diagnosis of apathetic thyroid storm secondary to uncontrolled Graves' disease. She was managed medically for thyroid storm with hopes to control the tachyarrhythmia by controlling the underlying etiology. Subsequently, her symptoms resolved, and she came back to baseline except for continued atrial fibrillation, which was rate controlled. Early recognition of an apathetic thyroid storm can prevent mortality and morbidity as it can often be missed due to atypical symptoms.

## Introduction

A thyroid storm is a complication of thyrotoxicosis. It has a high mortality rate of up to 30% if left untreated and is characterized by tachycardia or tachyarrhythmias, thermal dysregulation, and altered mental status. Thyroid storm may have an apathetic presentation, but this can either be missed or is underreported [1]. Lahey first described apathetic hyperthyroidism in 1931, with the main clinical characteristics being fatigue, weakness, apathy, and depression [2]. Atrial fibrillation as a presenting symptom for apathetic hyperthyroidism-related thyroid storm has rarely been reported in the literature. This is the first case with atrial fibrillation as an initial presentation of an apathetic hyperthyroidism-related thyroid storm to the best of our knowledge.

## Case presentation

**Figure 1:**
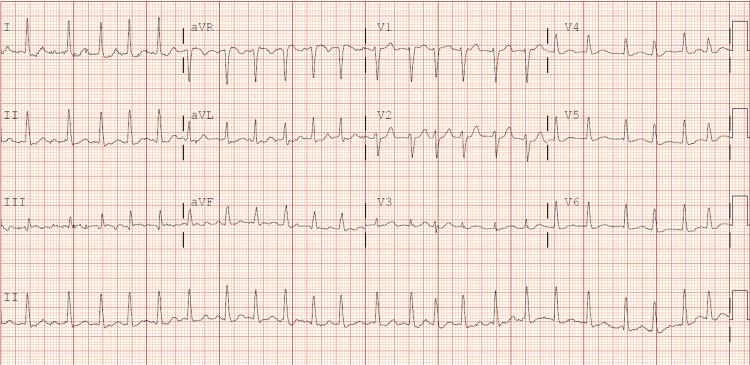
ECG showing irregular rhythm without P waves, consistent with a diagnosis of atrial fibrillation ECG - electrocardiogram

A 65-year-old female presented with a complaint of fatigue for the last two weeks. Her past medical history is significant for type II diabetes and peripheral arterial disease. The patient complained of generalized tiredness, poor appetite, nausea, and vomiting with low-grade fever. She denied heat intolerance, tremors, palpitations, or increased perspiration. In the emergency department, she was tachycardiac to 180s, and an electrocardiogram (ECG) showed atrial fibrillation with rapid ventricular rate (Figure 1). Her physical exam was negative for graves' ophthalmopathy, pretibial myxedema, or thyroid acropachy. Her neurological exam showed bradyphrenia. Initially, she was started on a diltiazem drip by cardiology as a bedside echocardiogram revealed an ejection fraction of more than 50%. On returning basic labs, significant findings included a thyroid-stimulating hormone (TSH) of less than 0.01 and elevated free T4 of 3.77. The free T3 was also elevated > 20. The TSH one month ago was within normal limits. No new medications were added to her regimen except semaglutide. Diltiazem drip was stopped, and endocrinology was consulted, and her Burch-Wartofsky Point Scale (BWPS) for thyrotoxicosis score was greater than 45, highly suggestive of thyroid storm. On the endocrinologist's recommendation, the patient was started on propylthiouracil, hydrocortisone, potassium iodide drops, and propranolol. The patient was later transitioned to methimazole.

The patient continued to remain in atrial fibrillation, which was rate controlled. The patient was also started on apixaban for anticoagulation. Her subsequent echocardiogram showed new-onset heart failure with a reduced ejection fraction of 35 to 40% and left atrial dilation (Video 1). The patient underwent a thyroid ultrasound which revealed a multinodular goiter with a dominant nodule in the isthmus. Her autoimmune work-up showed a positive thyroid receptor antibody test. Her thyroid-stimulating immunoglobulins were also elevated to 506%. Cardiology deferred direct current cardioversion (DCCV), and the patient was scheduled for an outpatient radioiodine thyroid uptake scan with the possible need of a fine needle aspiration (FNA) biopsy for further evaluation. A follow-up echocardiogram in two months did not show any decline of ejection fraction, and the patient was medically managed for hyperthyroidism and atrial fibrillation with rate control.

**Video 1:** Atrial fibrillation with reduced ejection fraction and left atrial dilation

## Discussion

Hyperthyroidism can present as an acute exacerbation with symptoms of thyrotoxicosis resulting in acute clinical deterioration (thyroid storm) [3]. A thyroid storm refers to the inability of a patient to maintain adequate metabolic, cardiovascular, or thermoregulatory control. BWPS for thyrotoxicosis is now widely used to predict biochemical thyrotoxicosis's likelihood of thyroid storm [4]. A BWPS score of ≥45 is highly suggestive of a thyroid storm, 25-44 of an impending thyroid storm, and a score of < 25 is unlikely to be a thyroid storm [2]. In an apathetic thyroid storm, the presentation of apathy is associated with weight loss, dry skin, tachyarrhythmias, and congestive cardiac failure [5]. Typical features of Graves’ disease, like hand tremor and ophthalmopathy, may be absent, as in our case, and physical exam may be unrevealing [5].

In general, the pathophysiology behind apathy is not well known. It has been hypothesized that lack of typical hyperthyroidism symptoms could be secondary to decreased circulatory amines (catecholamines) or depletion of the body's stores of amines. It could also be secondary to the inability of the body at the receptor level to respond to these amines [5]. Interestingly, a paradoxical improvement was observed when adrenergic blockade in patients with the apathetic crisis was employed [5]. Thyrotropin-releasing hormone (TRH) has an antidepressant effect when administered to severely depressed euthyroid patients [6]. This could be either due to increasing dopaminergic receptor sensitivity or altering the metabolism of dopamine in such a way as to increase the concentration of active dopamine in the brain. TRH has also been shown to reinforce the behavioral effects of levodopa in mice and is equally active in hypophysectomized animals [7]. However, whether it is depletion of TRH or catecholamines or receptor unresponsiveness, overlap with senile dementia could also mask the symptoms.

Although atrial fibrillation is a common cardiac complication of hyperthyroidism, it has been underreported as a presenting symptom of apathetic hyperthyroidism or a herald of thyroid storm in cases of apathetic hyperthyroidism. The incidence of atrial fibrillation also increases with advancing age [8]. Effects of thyroid hormones on ion channels of atrial myocytes contribute to the development of atrial fibrillation. Hyperthyroidism can result in shortened action potential duration, resulting in a higher inclination for the heart to go in atrial fibrillation [9]. Despite having an atypical presentation, thyroid storm associated with apathetic hyperthyroidism has increased T4/T3 activity leading to activation of these atrial myocytes and atrial fibrillation. Because of the apathetic symptoms, especially in the elderly, the diagnosis of hyperthyroidism is often overlooked, and only atrial fibrillation itself could be the presenting symptom, as in our case. The patients may have no prior history of hyperthyroidism.

## Conclusions

Our case highlights the occurrence of tachyarrhythmias, particularly atrial fibrillation, as an initial presenting symptom of an Apathetic thyroid storm in the setting of undiagnosed hyperthyroidism. Previously atrial fibrillation has been observed in conjunction with thyrotoxicosis, and the effect of thyroid hormone-mediated atrial myocyte stimulation is the reason behind it. Nausea, vomiting, and lethargy are directly related to apathetic thyrotoxicosis and can mask underlying hyperthyroidism, as these are not typical presentations. Atrial fibrillation is usually resolved after underlying thyroid function normalizes. The recognition of an apathetic thyroid storm is essential and can present as a diagnostic challenge.
